# Quick Returns: A Quasi‐Experimental Field Study on the Effects on Sleep, Fatigue and Cognitive Performance

**DOI:** 10.1111/jsr.70244

**Published:** 2025-12-15

**Authors:** Kristin Öster, Marie Söderström, Philip Tucker, John Axelsson, Göran Kecklund, Anna Dahlgren

**Affiliations:** ^1^ The Division of Psychology, Department of Clinical Neuroscience Karolinska Institutet Solna Sweden; ^2^ Department of Psychology Stockholm University Stockholm Sweden; ^3^ Stress Research Institute, Department of Psychology Stockholm University Stockholm Sweden; ^4^ School of Psychology Swansea University Swansea UK

**Keywords:** backward rotation, recovery, safety, short rest periods, sleep deprivation

## Abstract

As sleep restriction has negative effects on performance, ensuring sufficient sleep for shift workers is essential. Quick returns (< 11 h off between shifts) shorten sleep and are associated with increased fatigue and risk of accidents, but there is limited research on other aspects of cognitive performance and work performance. The aim of the present quasi‐experimental field study was to investigate the effects of quick returns on objective and subjective measures of sleep, fatigue and cognitive performance. In total 36 newly graduated nurses were followed during two pre‐scheduled work periods, with and without a quick return (evening–day–day vs. day–day–day). They kept diaries of sleep and work, wore actigraphy wristbands to record sleep and performed 3 × 3 min smartphone‐based cognitive tests (simple reaction time, episodic memory and Stroop) several times daily. Quick returns were found to shorten sleep by 46 min on average, and participants felt less rested in the morning and sleepier throughout the day. Sleep fragmentation and sleep efficiency did not differ between conditions but participants reported poorer sleep quality. Although the nurses reported cognitive impairments after a quick return, the estimated effects on simple attention, episodic memory and Stroop were small and overlapped zero. There were also indications of lingering fatigue on the second day shift after a quick return, but estimates are uncertain. In sum, quick returns shorten sleep and decrease subjective alertness, which could contribute to increased fatigue‐related risk at work, but people seem able to mobilise necessary resources to maintain performance on short cognitive tasks.

## 1 Introduction

Sleep is an essential form of recovery that enables us to remain alert and perform at work. Performance declines and fatigue increases as a function of time awake and is restored again after a full night of sleep (Åkerstedt et al. 2009). With no sleep or shortened sleep, alertness levels are not properly restored which may affect performance and safety. Thus, ensuring sufficient sleep for shift workers is important (Kecklund and Axelsson 2016).

To secure sufficient time for recovery and sleep in shift work, guidelines recommend that quick returns (< 11 h off between shifts) should be avoided (International Labour Office 2019). Quick returns between evening and day shifts are associated with a reduction in sleep duration to around 5–6 h (Vedaa et al. 2016), and with increased fatigue the following workday (Holmelid et al. 2024; Öster et al. 2023, 2024; van de Ven et al. 2021; Vedaa et al. 2016, 2017). Quick returns have also been associated with poorer subjective sleep quality (Holmelid et al. 2025; Öster et al. 2024), but these findings have not yet been corroborated in objective measures (Axelsson et al. 2004; Holmelid et al. 2025; Öster et al. 2024). Despite these negative effects and legislation against quick returns, one in five European workers report exposure to quick returns (Eurofound 2017).

Research on safety consequences of quick returns further indicates an increased risk of accidents and mistakes. Shift workers who work many quick returns report more accidents, near accidents and dozing off (Vedaa et al. 2019). Increasing the exposure to quick returns from one year to another, has also been associated with a corresponding increase in retrospectively reported accidents (Vedaa et al. 2020). Some studies on registry data have associated quick returns with an increased risk of injuries (Nielsen, Hansen, et al. 2019) and work accidents (Nielsen, Dyreborg, et al. 2019), but not all (Härmä et al. 2020). However, there is limited research on other aspects of cognitive performance and work performance.

Partial sleep restriction is associated with impaired cognitive performance, leading to slower responses and reduced accuracy in attention, executive function and memory tasks. The less sleep people get, and the more days with restricted sleep, the greater the decline (Lowe et al. 2017). Complex executive functions are also affected (Lowe et al. 2017; Wickens et al. 2015). This is in line with research on full sleep deprivation, where individuals become less flexible and creative in their thinking, and have difficulties maintaining concentration, filtering distractions, inhibiting responses and switching strategies (Horne 2012; Killgore 2010). However, performance on rule‐based or routine tasks appears largely unaffected even after no sleep (Killgore 2010). As quick returns limit sleep, they may compromise vigilance and attention and reduce the ability to handle unexpected situations, even if routine job performance remains intact.

It is also of interest to investigate the daily variations in the ability to maintain performance and alertness after a quick return, to determine if and when fatigue‐related risk is elevated. Alertness (Åkerstedt and Folkard 1997), cognitive performance (Carrier and Monk 2000) and the vulnerability to sleep loss (Hudson et al. 2020; Van Dongen and Belenky 2009) are all known to vary across the day as a function of homeostatic sleep pressure (high pressure reduces performance and increases fatigue) and circadian timing (e.g., poor performance and elevated fatigue in the early morning hours). Theories on sleep–wake regulation propose that sleep loss induces more attentional problems in the early morning with improvements in the afternoon (Hudson et al. 2020; Van Dongen and Belenky 2009), although data from experimental sleep loss studies sometimes have failed to show such time‐of‐day effects (Holding et al. 2021).

A few field and lab studies have examined but failed to find effects of quick returns on cognitive performance. However, design limitations hinder firm conclusions. In an intervention study, simple attention during 8 h forward‐rotating shifts with quick returns, did not differ from 12 h shifts without quick returns (Lowden et al. 1998). However, 12 h shifts may also induce fatigue, which may have biased the comparison, and factors relating to the intervention could also have affected the results. Another study found no differences in simple attention across shifts in a forward‐rotating schedule with quick returns (night–evening–morning) (Axelsson et al. 2004), but lacked a comparison with longer rest periods. A well‐controlled lab study simulating quick returns and day transitions also found no effects on cognition, but lacked the workload intensity of jobs like nursing, potentially affecting the outcomes (Holmelid et al. 2024). Overall, there is a lack of field studies comparing evening–day and day–day transitions under controlled conditions.

On top of acute effects, there is some evidence that quick returns could increase fatigue for several days, at least in shift systems with consecutive early day shifts that hinder proper recovery (Lowden et al. 1998). The possibility of such lingering effects matters not only for safety but also for study design. In diary studies comparing evening‐day and day‐day transitions, failing to account for the previous shift could mask true effects due to residual fatigue. For instance, both conditions may inadvertently be drawn from a single evening‐day‐day‐…‐ sequence (Öster et al. 2024). This highlights the need for field studies with rigorous experimental control to accurately assess both the acute and lingering consequences of quick returns on fatigue‐related risks.

**FIGURE 1:**
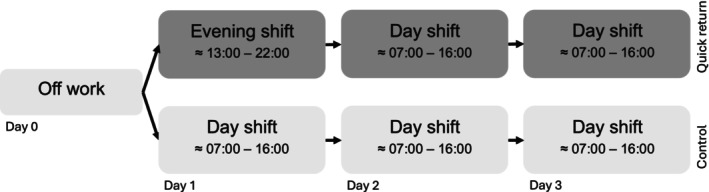
Quasi‐experimental design.

The primary aim of the current quasi‐experimental field study was to estimate the acute effects of quick returns (evening‐to‐day shift transitions) compared to day‐to‐day shift transitions on objective and subjective measures of sleep, fatigue and cognitive performance. We hypothesized that quick returns would result in shortened sleep duration (Hypothesis 1), reduced sleep quality (Hypothesis 2), reduced cognitive performance (Hypothesis 3), increased fatigue (Hypothesis 4) and increased stress at bedtime (Hypothesis 5). Secondly, we wanted to investigate the existence of lingering fatigue and performance deficits on the second day shift after a quick return (see Figure 1). We hypothesized that there would be spillover fatigue (Hypothesis 6) and lingering reductions in cognitive performance (Hypothesis 7). Lastly, we wanted to explore if the effect of quick returns on fatigue and objective cognitive performance varied with time of day.

Hypothesis 1 Quick returns result in shortened sleep duration.

Hypothesis 2 Quick returns result in reduced sleep quality.

Hypothesis 3 Quick returns result in reduced cognitive performance.

Hypothesis 4 Quick returns result in increased fatigue.

Hypothesis 5 Quick returns result in increased stress at bedtime.

Hypothesis 6 Quick returns result in lingering fatigue on the second day shift.

Hypothesis 7 Quick returns result in lingering reductions in cognitive performance on the second day shift.

## 2 Materials and Methods

### 2.1 Study Population and Experimental Design

Between 2018 and 2023, newly graduated nurses were recruited from introductory programs at three hospitals. The data collection was paused from March 2020 to October 2021 due to the COVID‐19 pandemic. Of 88 signing up, 60 met eligibility criteria and provided informed consent. Inclusion required less than 2 years of nursing experience upon entering the study and a schedule allowing quick returns. Exclusion criteria included diagnosed sleep disorders and use of sleep medication (except melatonin). Participants avoiding quick returns due to medical conditions or long commutes were also excluded, as it was deemed unethical to ask them to work quick returns. The two‐year cut‐off was chosen to minimise selection bias due to the ‘healthy worker effect’, wherein more vulnerable or less healthy individuals tend to leave shift work, potentially causing an underestimation of the true effect size (Knutsson 2004). Ethical approval was granted by the Swedish Ethical Review Authority (dnr: 2018/1541‐31).

The study was designed to follow each participant during one quick return (off‐evening‐**day**‐day) and one control condition (off‐day‐**day**‐day); see Figure 1. Both conditions were preceded by a day off work (off) to control for spillover fatigue. The main analysis concerned acute effects of quick returns on the second shift (marked in bold). The third shift in both sequences was added to assess the presence of lingering effects. The order of the shift sequences was randomised across participants.

The participants were asked to find the shift sequences in their existing schedule. If there were no occurrences, they were asked to switch shifts with a colleague or to ask for the shift sequences in their upcoming scheduling period. In total, 35 participants managed to schedule and provide data from both conditions.

### 2.2 Measurements

#### 2.2.1 Sleep and Fatigue

Participants kept a diary of sleep and work for 3 days during each shift condition, starting on day 1 (experimental condition, see Figure 1). Starting on the evening of day 0 (baseline), participants wore an actigraphy watch during sleep for the three nights prior to each shift.

Sleep duration was assessed with actigraphy data on assumed sleep time, defined as the total elapsed time between falling asleep and waking up. Sleep quality was assessed with actigraphy data on sleep fragmentation (percentage of mobile time and immobile bouts ≤ 1 min during sleep), sleep efficiency (percentage of time asleep between falling asleep and wake‐up) ratings on feeling rested (1 = very, 5 = completely), and the Karolinska Sleep Diary—Sleep Quality index (KSD‐SQI) (Keklund and Åkerstedt 1997). Participants also rated their level of anxiousness at bedtime (1 = very, 5 = not at all). The baseline questionnaire included items on habitual sleep need and diurnal type (1 = pronounced morning type, 5 = pronounced evening type).

Participants rated their fatigue level on the Karolinska sleepiness scale (KSS), a 9‐point Likert scale ranging from 1 (‘extremely alert’) to 9 (‘extremely sleepy, fighting sleep’) (Akerstedt and Gillberg 1990) every third hour from 07:00 until bedtime. The diary also included an item on workload (1 = very high, 5 = very low).

#### 2.2.2 Cognitive Tests

Participants were instructed to perform a smartphone‐based cognitive test battery three times across the day: in the morning (06:00–10:00), afternoon (12:00–17:00) and evening (≥ 18:30, not episodic memory) on the second and third day of each condition (see Figure 1). For more information on the cognitive tests, see Holding et al. (2021).

Simple attention was assessed with a three‐minute single choice reaction time test. Response times below 150 ms (slightly longer than the typical 100 ms due to touch screen latency) and above 3000 ms were removed. Due to touch screen latency, using a threshold to signify a lapse would have been inappropriate (Holding et al. 2021). Instead, lapses were defined as twice the individual mean response time (Basner and Dinges 2011).

To assess episodic memory, participants were instructed to memorise a list of 12 words (presented for 12 s, i.e., the encoding phase). Then, after a delay of 5 s, they had to identify the presented words among a list of 24 words (also including 12 dummy words). This recognition phase was self‐paced. The encoding and recognition phases were then repeated using the same 12‐word list and 12 new dummy words. This resulted in 48 responses, and the analyses focused on the probability of incorrect recognition (i.e., incorrectly identifying a dummy word, or failure to identify a previously shown word).

Cognitive inhibition was assessed using a three‐minute Stroop test. We were interested in response times during incongruent and congruent trials, the probability of mistakes (i.e., naming the meaning of the word, instead of the font colour) and the difference in response time between congruent/incongruent trials and congruent/congruent trials (i.e., the congruency effect). See Holding et al. (2021) for more details. Responses > 3000 ms and < 500 ms were removed.

To reduce learning effects, participants were instructed to complete two practice sessions before data collection: first, with a researcher during a preparatory phone call, and then once more.

#### 2.2.3 Subjective Cognitive Ability

In the work diaries, participants were asked to rate their ability to make decisions; see the overall picture; mentally keep track of things to do; perform work safely; and be present when interacting with others; on Likert scales ranging from 1 (‘excellent’) to 5 (‘very bad’). The five items were reduced to a single composite measure using principal component analysis, with the R Stats Package (R Core Team 2022).

### 2.3 Statistical Analysis

Data were analysed in the R programming language, using Bayesian linear mixed models with *rstanarm* package (Goodrich et al. 2022). Advantages of a Bayesian approach include greater flexibility in model specification (e.g., maximal random structures; Barr et al. 2013), as the models utilise Markov Chain Monte Carlo (MCMC) simulation. Bayesian models yield probability distributions for parameter estimates, which align with intuitive interpretations of uncertainty (McElreath 2020). Thereby, the uncertainty for any desired outcome (means, effect sizes, standard deviations etc.) can be obtained directly from the posterior distribution. The probability distribution is summarised as 95% compatibility intervals (CIs) using the *rethinking* package (McElreath 2023), which contain the equivalent range of parameter estimates compatible with the data and model assumptions. The 95% threshold was chosen to parallel the conventional *α* = 0.05. MCMC‐convergence was verified via trace plots, $\hat{R}$ and the effective sample sizes.

Primary analyses focused on day two, i.e., acute effects of quick returns. Primary outcomes were sleep duration, simple attention response time, probability of misremembering words, and Stroop incongruent trials response times. Secondary outcomes included sleep fragmentation index, sleep efficiency, sleep quality index, bedtime anxiousness, feeling rested upon awakening, subjective cognitive ability, attention lapses, Stroop congruent trials, cognitive conflict and Stroop test mistakes. Lingering effects on day three were assessed for fatigue, feeling rested and cognitive performance. For outcomes measured multiple times per day, time‐of‐day interactions were included as secondary analyses.

#### 2.3.1 Model Specifications and Prior Probability Distribution

Data on lapses and mistakes were modelled in binomial models using logit links. All other models assumed a Gaussian distribution. Response times were expressed as 1/s, a metric known to be sensitive to sleep deprivation (Basner and Dinges 2011). Full details on model specifications and priors are available in the Supporting Information.

We used weakly regularising priors to reduce the influence of extreme data points on parameter estimates. For the effect of quick returns, priors were centred at no expected difference between conditions. The width of the prior probability interval was based on our previous effect estimates (Öster et al. 2024) and the work of Holding et al. (2021), and adjusted when needed for prior predictions to stay within the scale limits. For response times, we applied rstanarm's default priors. In simpler terms: Before analysing the data, we assumed that there would be no difference between conditions, and if a difference did exist (in either direction), the effect size was expected to be in comparison with earlier findings. These models offer a more conservative approach than maximum likelihood models.

#### 2.3.2 Pre‐Registration

The analysis plan, which specified primary and secondary outcomes, cut‐offs for outliers, model specifications and prior predictive checks, was pre‐registered on the OSF platform prior to accessing the full dataset: https://osf.io/kr4su. Deviations from the pre‐registration are listed in the Supporting Information.

## 3 Results

Mean estimates, 95% compatibility intervals (CI) and model convergence metrics for primary and secondary outcomes are presented in Table 1.

**TABLE 1:** Estimated means, estimated difference between conditions, Cohen's D (for gaussian models) and odds ratios (for binomial models).

	*N* ^a^	Mean		Mean difference (95% CI^b^)	$\hat{R}$	*N* (eff)^c^	Cohen's D (95% CI^b^)	Odds ratio
		Control	QR					
Primary outcomes								
Sleep duration, hours: minutes	36	7:00	6:13	−46 min (−68, −26)	1	5965	−1.03 (−1.56,‐0.51)	
Sleepiness, 1–9 very sleepy	36	4.76	5.19	0.43 (0.09, 0.77)	1	1376	0.37 (0.08, 0.67)	
Simple attention, ms	34	449.75	458.26	9 (−3, 20)	1	966	0.11 (−0.04, 0.24)	
Episodic memory, probability (*p*)	35	0.08	0.08	0.00 (−0.02, 0.02)	1	2757		1.05
Incongruent Stroop trials, ms	35	1055.10	1074.95	20 (−21, 62)	1	997	0.07 (−0.08, 0.22)	
Secondary outcomes								
Fragmentation index, %	36	21.42	21.77	0.35 (−3.75, 4.45)	1	6287	0.04 (−0.40, 0.47)	
Sleep efficiency, %	36	88.98	88.50	−0.48 (−1.64, 0.71)	1	4704	−0.19 (−0.66, 0.27)	
Sleep quality, 1–5 high	36	4.08	3.38	−0.70 (−1.08, −0.34)	1	7439	−0.92 (−1.44, −0.41)	
Anxiousness at bedtime, 1–5 low	36	4.42	3.26	−1.16 (−1.51, −0.78)	1	4543	−1.47 (−2.10, −0.86)	
Feeling rested, 1–5 high	36	2.57	1.88	−0.70 (−1.02, −0.37)	1	6100	−0.98 (−1.53, −0.48)	
Sleepiness at work, 1–9 very sleepy	36	4.69	5.27	0.58 (0.18, 0.99)	1	3007	0.55 (0.17, 0.95)	
Cognitive ability^d^, −8–3 excellent	34	0.46	−0.48	−0.95 (−1.73, −0.17)	1	5950	−0.59 (−1.10, −0.10)	
Attention lapses, probability (*p*)	34	0.01	0.01	0.00 (0.00, 0.01)	1	2225		1.76
Stroop mistakes, probability (*p*)	35			0.00 (−0.02, 0.01)	1	1053		0.94
Congruent Stroop trials, ms	35	955	968	13 (−17, 43)	1	1501	0.06 (−0.08, 0.19)	
Cognitive conflict, ms	35	−28.47	−12.42	−16 (−34, 1)	1	19,168	−0.19 (−0.27, −0.11)	
Lingering effects on day 3								
Sleep duration, hours: minutes	35	7:09	7:14	0.05 (−0.44, 0.53)	1	6054	0.05 (−0.42, 0.51)	
Feeling rested, 1–5 high	35			−0.34 (−0.68, 0.01)	1	6294	−0.45 (−0.92, 0.02)	
Sleepiness at work, 1–9 very sleepy	34	4.86	5.29	0.43 (−0.09, 0.96)	1	4698	0.43 (−0.09, 0.96)	
Simple attention, ms	32	467.91	480.04	12 (−5, 30)	1	1299	0.13 (−0.05, 0.31)	
Episodic memory, probability (*p*)	33	0.09	0.09	0.00 (−0.03, 0.03)	1	2370		1.01
Incongruent Stroop trials, ms	33	994.84	1021.26	26 (−1, 55)	1	1462	0.10 (0.00, 0.21)	
Cognitive ability, −8–3 excellent	33	0.38	−0.32	−0.70 (−1.59, 0.17)	1	5572	−0.39 (−0.89, 0.09)	
Attention lapses, probability (*p*)	32	0.01	0.02	0.01 (0.00, 0.01)	1	2294		1.46
Stroop mistakes, probability (*p*)	33	0.04	0.03	−0.01 (−0.02, 0.01)	1	1282		0.90
Congruent Stroop trials, ms	33	903.13	917.58	14 (−7, 37)	1	964	0.07 (−0.04, 0.18)	
Cognitive conflict, ms	33	17.27	16.00	1 (−19, 21)	1	9826	−0.17 (−0.25, −0.08)	

Abbreviations: Control, day–day; QR, Quick return, evening–day.

^a^ Number of participants with complete data for the variable of interest and thus included in the analysis.

^b^ CI, compatibility intervals.

^c^ Effective sample size, a measure of MCMC convergence.

^d^ A change of 0.95 on this index variable corresponds to rating one item 1.7 scale steps worse.

### 3.1 Descriptives

The participants had worked on average 7 (SD = 4) months in their current position; 86% were women. All but one worked full‐time. Commuting times averaged 16 min (SD = 8), and the perceived workload was rated as fairly high (Mean = 2.6, SD = 1; scale: 1 = very high, 5 = very low). Average shift times were 12:48–21:46 for evening shifts, and 6:48–15:41 for day shifts. The time off during quick returns averaged 9 h (SD = 28 min). Most participants (75%) reported working at least one quick return per week in the past month, one reported an occasional quick return, and the rest had 2–3. A majority (75%) considered quick returns a major problem. One in five participants rated themselves as neither morning nor evening types. There was an evening distribution of pronounced evening and morning types (19% and 17%, respectively) but a slight overrepresentation of ‘somewhat morning types’ (31%) to ‘somewhat evening types’ (14%). The average habitual sleep need ranged from 5 to 9 h, with an average of 7 h 36 min.

### 3.2 Sleep and Fatigue

Nurses were estimated to sleep on average 7 h (95% CI [6 h 41 min, 7 h 19 min]) during day transitions, and 47 min (95% CI [1 h 8 min, 26 min]) shorter during a quick return. The night after a quick return, that is the night between day 2 and 3, the sleep duration did not differ between conditions (0.05, 95% CI [−0.44, 0.53]).

The subjective sleep quality index was 4.08 (95% CI [3.8, 4.38]) on average during day–day transitions and −0.7 units lower during quick returns (95% CI [−1.08, −0.34]). Nurses were also estimated to feel 1.16 (95% CI [0.78, 1.51]) scale units more anxious at bedtime on average during quick returns compared to day transitions. However, the degree of sleep fragmentation and sleep efficiency did not differ between conditions (95% CI [−3.75, 4.45] and [−1.6, 0.7], respectively). The average degree of sleep fragmentation was 21% (95% CI [18, 24]) during day transitions, and the estimated sleep efficiency was 89% (95% CI [88, 90]).

In the morning after a quick return, nurses rated themselves as feeling −0.7 (95% CI [−1.02, −0.37]) scale points less rested upon wakening, compared to day transitions. The effect lingered on the third morning (−0.34), but the compatibility interval overlapped no effect (95% CI [−0.68, 0.01]). The posterior probability of a lingering effect larger than 0.2 scale points was 79% (*P*(difference in feeling rested < −0.2 | data = 0.79)).

The average sleepiness during work hours (07:00–16:00) was estimated to be 4.69 (95% CI [4.22, 5.17]) during day–day transitions and 0.58 (95% CI [0.18, 0.99]) scale units higher during quick returns, resulting in a mean sleepiness of 5.27 (95% CI [4.76, 5.78]). Seen across the entire day (07:00–22:00) the estimated difference was 0.43 (95% CI [0.09, 0.77]) scale points. On the third day of the quick return condition, nurses were still estimated to be 0.43 scale points sleepier compared to the control, but estimates of no change were also plausible (95% CI [−0.09, 0.96]). The posterior probability that nurses are at least 0.2 scale points sleepier on the third day was 82% (*P*(difference in sleepiness > 0.2 | data) = 0.82).

**FIGURE 2:**
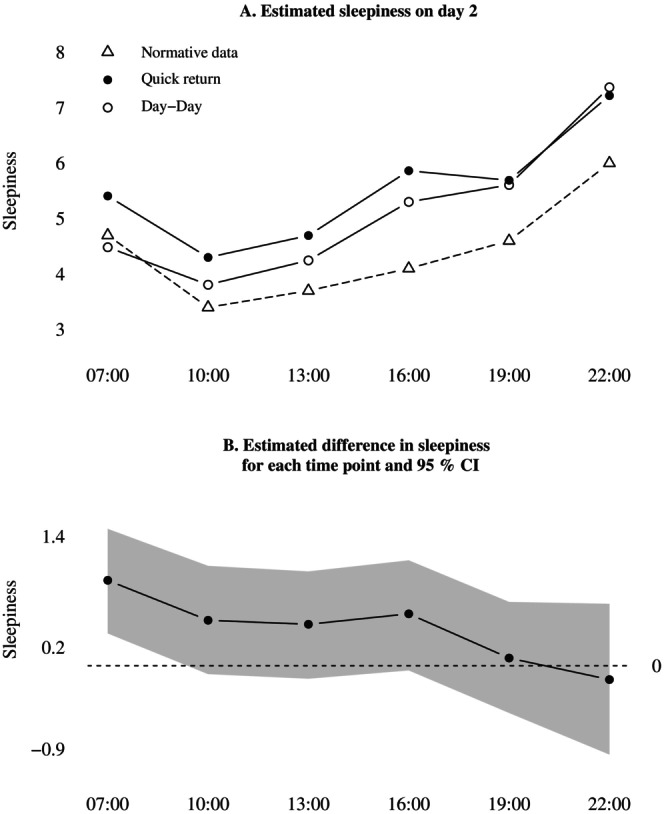
Estimated interaction between shift condition and time of day, for sleepiness on day 2. In plot A, the estimated mean sleepiness levels are plotted against normative data from Åkerstedt et al. (2017). (A) Estimated sleepiness on day 2. (B) Estimated difference in sleepiness for each time point and 95% CI.

As a secondary analysis of sleepiness on the second day of the quick return, we added an interaction term between quick returns and time of day. The variation in sleepiness across the second day is displayed in Figure 2A. Adding the interaction term increased the uncertainty of estimates, whereby the only reliable increase in sleepiness was found at 07:00; see Figure 2B. At 10:00–16:00, nurses were also estimated to be sleepier during quick returns, but the compatibility interval spanned effects on no difference. At 19:00 and 22:00, the mean estimate was no difference between conditions.

### 3.3 Cognitive Tests

On the second day of a quick return, simple attention response times were estimated to be 9 ms slower compared to day transitions, but the compatibility interval overlapped zero (95% CI [−3, 20] ms). On the third day, the mean response times were 10 ms slower, but again, the compatibility interval overlapped zero (95% CI [−5, 30] ms). No reliable increase in lapse risk was observed on either the second (95% CI [−0.002, 0.012]) or third day (95% CI [−0.002, 0.014]).

**FIGURE 3:**
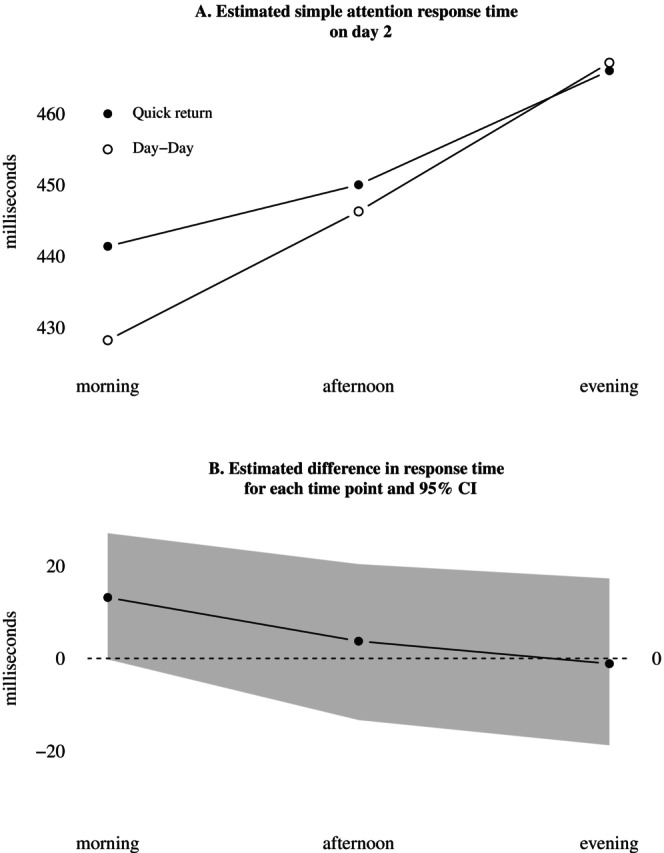
Estimated interaction between shift condition and time of day, for simple attention response times on day 2. (A) Estimated simple attention response time on day 2. (B) Estimated difference in response time for each time point and 95% CI.

Figure 3A displays the estimated interaction between quick returns and time of day for response times on day 2. The largest mean difference (13 ms) appeared in the morning, but estimates of no difference were also compatible with data (95% CI [0, 27]). As is evident from Figure 3B, the mean estimated difference was close to zero in the afternoon and evening, but included much uncertainty.

During incongruent Stroop trials, nurses responded on average 20 ms slower on the second day of a quick return, but estimates of shorter response times were also compatible with the data (95% CI [−21, 62] ms). Likewise, there was no reliable difference on the third day (95% CI [−1, 55] ms). No reliable differences were found for secondary outcomes (congruent trials, probability of mistakes, congruency effect) on either day (see Table 1).

**FIGURE 4:**
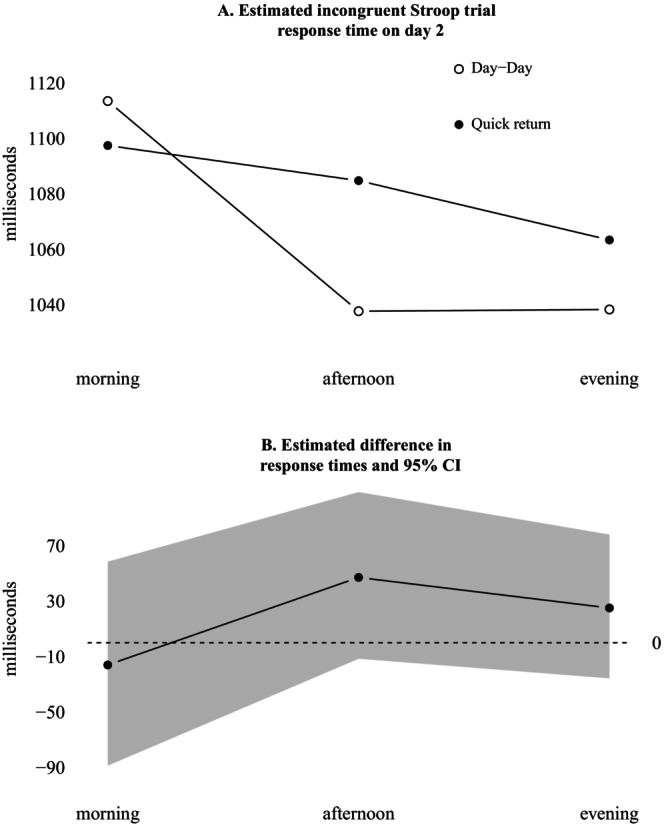
Estimated interaction between shift condition and time of day, for response times during incongruent Stroop trials on day 2. (A) Estimated incongruent Stroop trial response time on day 2. (B) Estimated difference in response times and 95% CI.

Figure 4A displays the estimated interaction between shift condition and time of day for incongruent Stroop trials on day 2. During day transitions, response times improved in the afternoon (95% CI [−115, −37]ms) and evening (95% CI [−123, −29] ms) compared to the morning. This improvement was not seen during quick returns (95% CI [−84, 56] and [−92, 21], respectively). However, response times cannot be said to differ between conditions with 95% certainty, as the compatibility interval spanned estimates of no change for all time points (see Figure 4B).

**FIGURE 5:**
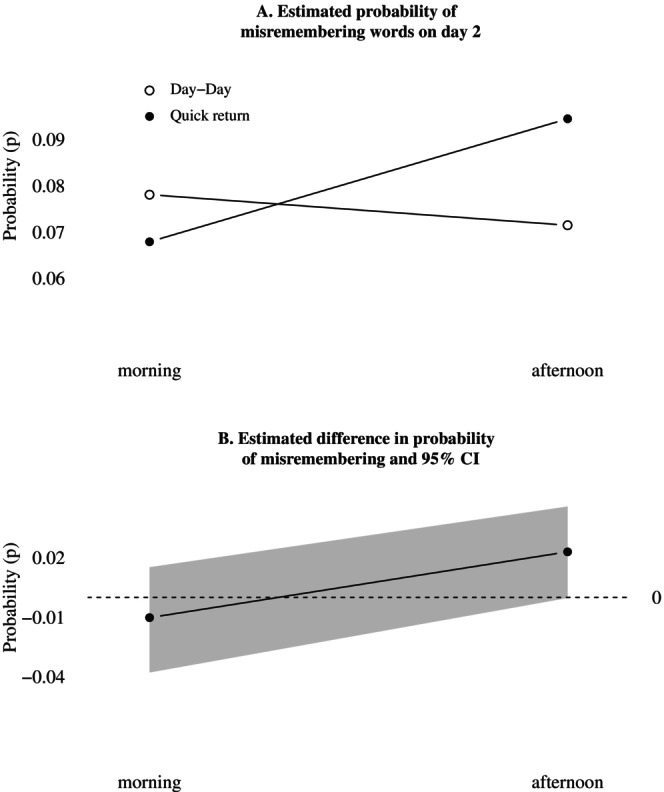
Estimated interaction between shift condition and time of day, for the probability of misremembering words on day 2. (A) Estimated probability of misremembering words on day 2. (B) Estimated difference in probability of misremembering and 95% CI.

The average probability of misremembering a word on the second day of a quick return was 0.08 (95% CI [0.07, 0.09]) and did not deviate from day transitions on either day 2 (95% CI [−0.02, 0.02]) or day 3 (95% CI [−0.02, 0.03]). Figure 5A displays the interaction between shift and time of day for day 2, which indicated a 3 percentage point (95% CI [0.004, 0.049]) increase in the probability of misremembering words in the afternoon compared to the morning during quick returns. When comparing quick returns to day transitions, the estimated difference was also 3 (95% CI [0.001, 0.067]) percentage points larger and in the opposite direction in the afternoon, but the estimated difference in the afternoon was not statistically reliable as the compatibility interval includes zero (see Figure 5B).

### 3.4 Subjective Cognitive Ability

During quick returns, nurses experience a reduction in cognitive ability during work (−0.95, 95% CI [−1.73, −0.17]). On the third day, there was still a mean estimated reduction of cognitive ability (−0.7), but the compatibility interval contained estimates of no difference (95% CI [−1.59, 0.17]).

## 4 Discussion

In this experimental field study on newly graduated nurses with a fairly high workload, quick returns were found to shorten sleep to approximately 6 h, which manifests in increased daytime fatigue and subjectively reduced cognitive ability compared to day‐to‐day transitions. Quick returns did not result in more fragmented or less efficient sleep, but nurses experienced poorer sleep quality and felt less rested in the morning. Data from cognitive tests indicate that quick returns do not result in worse attention, short‐term memory, or inhibitory control—at least not on tests of short duration. Thus, the hypotheses related to objective sleep duration (Hypothesis 1), fatigue (Hypothesis 4), and bedtime stress (Hypothesis 5) were confirmed, whereas the hypotheses related to cognitive performance (Hypothesis 3) and sleep quality (Hypothesis 2) were supported in subjective measures but not objective.

Our findings are in line with previous studies utilising objective data: quick returns shorten sleep duration, reduce perceived sleep quality, but do not seem to result in objectively more fragmented sleep (Axelsson et al. 2004; Holmelid et al. 2025; Öster et al. 2024). The finding that nurses feel less rested in the morning is indicative that sleep during quick returns is nonetheless insufficient.

After a quick return, nurses reported more fatigue throughout the day, consistent with prior studies showing similar raw effect sizes (Holmelid et al. 2024; Öster et al. 2024; Vedaa et al. 2017). The mean sleepiness levels in both conditions were elevated compared to a normative sample of ~500 day workers, see Figure 2A. In the normative sample, worktime sleepiness levels mostly varied around ‘4—rather alert’ (Åkerstedt et al. 2017). In contrast, following a quick return nurses experienced sleepiness levels between 5 and 6 on average, meaning that they never experienced feeling alert for an entire workday. While a mean increase of 0.5 scale points may not be noticeable for the individual, its consistency with previous findings and the already elevated baseline suggest that it could contribute to increased fatigue‐related risks at the aggregated level.

Our study indicates that nurses perceive a slight reduction in their perceived cognitive ability, but do not perform worse on objective cognitive tests. Whereas the cognitive tests assess simple attention, short‐term memory and inhibitory control, the subjective items were designed to capture subjective ratings of more complex cognitive tasks. A possible interpretation of our findings is that nurses become mildly sleep deprived and slightly fatigued during quick returns which could contribute to a small increase in risk on complex work tasks, but that they can mobilise necessary resources to maintain performance on simple tasks of short duration. How this translates into potential safety risks needs to be addressed in future studies.

Our study cannot be taken as direct evidence that quick returns have no effect on cognitive functioning across the work shift, as estimates of increased response times, lapses and mistakes are also plausible given data. Further, by inspecting the upper bounds of the compatibility intervals, we can infer that nurses are likely to misremember at most 1 more word out of 48 words, lapse once more during 100 trials, react at most 20 ms slower during a test of simple attention and 62 ms slower during incongruent Stroop trials. Taken together, estimates of essentially no difference between conditions are most plausible given our data, and if quick returns affect performance on simple cognitive tests, the increase in risk is likely to be small, or take a longer time to become manifest.

On the third measurement day, participants averaged 7 h of sleep in both conditions, yet reported feeling more fatigued, less rested, and a reduced cognitive ability after the quick return, although estimates also allowed for no difference. The mean effect, though small and perhaps not noticeable to most individuals, indicates the possibility of incomplete recovery from the previous night, which could contribute to increased risks on an aggregated level. Although we cannot conclude lingering fatigue with 95% certainty, our model shows 80% confidence in an effect of at least 0.2 scale points. This aligns with previous findings of elevated fatigue persisting into subsequent day shifts following a quick return (Lowden et al. 1998). Together, these results offer tentative support for lingering effects (Hypothesis 6–7), warranting attention to the complete sequence in a block of shifts when comparing between shift transitions. They also highlight that having repeated morning shifts may not allow for full recovery, and if so, then quick returns should ideally be followed by no more than 1 day shift or possibly an evening shift.

The negative effects of partial sleep deprivation are known to accumulate for every additional day with sleep deprivation (Hudson et al. 2020; Killgore 2010; Wickens et al. 2015). Thus, although an occasional single quick return might not be a safety hazard, repeated quick returns could result in an accumulation of fatigue and thus increase the risk of fatigue‐related risks. Likewise, if quick returns are paired with other fatigue‐inducing factors, such as a heavy workload, long work hours, or frequent night shifts to name a few, the fatigue‐related risk could again accumulate. As our tests were brief and simple in nature, it is also uncertain how objective performance on extended and complex tasks would be affected. Future studies should investigate the effects of repeated quick returns on fatigue, cognitive performance, performance on complex tasks and potential safety consequences.

It would also be of interest to investigate individual differences in fatigue and performance deficits following quick returns, as there are known to be stable, individual differences in susceptibility to sleep loss (Hudson et al. 2020; Van Dongen et al. 2011). Short commuting times, a morning diurnal type, a short habitual sleep need and having short sleep onset latencies could facilitate longer sleep despite having short rest periods and thus increase tolerance. It would also be of interest to investigate factors that contribute to anxiety at bedtime and interfere with falling asleep. Finally, factors relating to the workplace, such as the workload or possibility for rest breaks, could also affect tolerance.

### 4.1 Strengths and Limitations

A key strength of this study is its quasi‐experimental design, enabling comparisons between quick returns and day‐to‐day shift transitions within otherwise equal settings. This also allowed us to not only control for spillover effects between shifts, but estimate the size of potential lingering fatigue. By focusing on newly graduated nurses, we also minimised selection bias linked to the “healthy worker effect”. However, the relatively small sample size limits the study's statistical power and interpretability of null effects. Participants' awareness of the study's purpose and their negative attitudes towards quick returns may have influenced their performance and ratings, though the representativeness of these attitudes is uncertain. Finally, it is not known whether participants compensated for sleep loss through behaviours like increased caffeine intake or more frequent rest breaks.

It is important to note that our comparison is between quick returns and day transitions where nurses' sleep is also likely to be curtailed. On day shifts, the nurses began work at 06:50 and slept 7 h on average. Thus, our data cannot be used to conclude how quick returns affect fatigue, performance and safety compared to ‘optimal’ working hours, for example a later start time of the day shift. Our study also lacks baseline data on prior sleep and how the participants would have performed when being well rested.

It could be argued that longer tests such as the standard 10 min PVT, would have increased the sensitivity of the study. However, the shorter 3 × 3 min tests were chosen to increase the feasibility of the study. It would have been difficult for the participants to find time for longer tests during their workday. Evaluation of the Karolinska wake app has shown that performance on even shorter—2‐min—tests is sensitive to sleep deprivation (Holding et al. 2021).

Although actigraphy data is considered more reliable than self‐reported sleep, and a more feasible substitute to polysomnography, it is known to overestimate total sleep duration and sleep efficiency measures as it underestimates time awake (Conley et al. 2019).

## 5 Conclusions

Our study suggests a slight increase in fatigue‐related risks after a single quick return, though people seem able to mobilise necessary resources to maintain performance on short cognitive tasks. Further, shift workers may risk elevated fatigue when consecutive dayshifts follow a quick return, but the estimates are uncertain. Future research should investigate how to ensure sufficient sleep following a quick return, as well as the impact on real work performance, safety implications and the risks from repeated quick returns.

## Author Contributions

Kristin Öster has collected the data, wrote the analysis plan and analysed the data. She has been the main author of the manuscript. Marie Söderström has been involved in the study design and contributed to the writing of the manuscript. Philip Tucker has contributed to writing the manuscript. John Axelsson has been involved in the study design and has contributed to both the analysis plan and the manuscript. Göran Kecklund has contributed to writing the manuscript. Anna Dahlgren is the principal investigator and has designed the study. She has been involved in the data collection, the analysis plan and in writing the manuscript.

## Funding

This work was supported by the Forskningsrådet om Hälsa, Arbetsliv och Välfärd (2017‐02032).

## Conflicts of Interest

The authors declare no conflicts of interest.

## Supporting information


**Data S1:** Supporting Information.

## Data Availability

Research data are not shared, as it was stated in the participant information that the data would not be made available to anyone outside the research group.
