# Supplementary material for: Quick Returns: A Quasi‐Experimental Field Study on the Effects on Sleep, Fatigue and Cognitive Performance
Source: J Sleep Res. 2025 Dec 15;35(3):e70244. doi: 10.1111/jsr.70244 (PMC13193494; doi:10.1111/jsr.70244)
Supplement: Supplementary file 1 — Data S1: Supporting Information. [file JSR-35-e70244-s001.docx]

Supplementary material

In this document, we list the model specifications and priors for the paper “Quick returns: a quasi-experimental field study on the effects of sleep, fatigue and cognitive performance”. Both the mathematical notation and stan_glmer formula used are provided. This document also contains a report of all deviations from the pre-registration for transparency.

# Model specifications and priors

Both the mathematical notation and stan_glmer formula used are provided.

## **Sleep length (assumed sleep)**

$$\begin{matrix} Likelihood & : \\ \mu_{i} & =\beta_{0}+u_{\text{[subj[i])}}+\beta_{1}\times\text{shift}_{i} \\ Sleep length & \sim Normal\left( \mu_{i},\sigma_{e} \right) \\ & Priors: \\ \beta_{0} & \sim Normal\left( 7,.5 \right) \\ \beta_{1} & \sim Normal\left( 0,1 \right) \\ u & \sim Normal\left( 0,\sigma_{u} \right) \\ \sigma_{e},\sigma_{u} & \sim Exponential\left( 1 \right) \\ & \end{matrix}$$

**Notes:**$\beta_{0}$ is the fixed-effects intercept,in this case the mean sleep length during day-day transitions
$\beta_{1}$ is the fixed-effects slope, in this case the difference in sleep length during quick returns compared to day-day transitions
$u$ is the by-subject varying intercepts, that is the each subject’s estimated variability around the fixed-effects intercept.

**rstanarm code:** stan_lmer(formula = assumed_sleep_h ~ QR_week + (1 | id), data = d1, cores = 4, refresh = 0, prior = normal(0, 1, autoscale = F), prior_intercept = normal(7, 0.5, autoscale = F), prior_aux = exponential(1, autoscale = F))

## **Fragmentation index**

$$\begin{matrix} Likelihood & : \\ \mu_{i} & =\beta_{0}+u_{\text{[subj[i])}}+\beta_{1}\times\text{shift}_{i} \\ Fragmentation index & \sim Normal\left( \mu_{i},\sigma_{e} \right) \\ Priors & : \\ \beta_{0} & \sim Normal\left( 24,10 \right) \\ \beta_{1} & \sim Normal\left( 0,7 \right) \\ u & \sim Normal\left( 0,\sigma_{u} \right) \\ \sigma_{e},\sigma_{u} & \sim Exponential\left( 0.1 \right) \\ & \end{matrix}$$

**rstanarm code:** stan_glmer(formula = fragmentation_index ~ QR_week + (1 | id), data = d1, family = gaussian, cores = 4, prior = normal(0, 7, autoscale = F), prior_intercept = normal(24, 10, autoscale = F), prior_aux = exponential(0.1))

## **Anxiousness at bedtime (orolig_sanggaendet)**

$$\begin{matrix} Likelihood & : \\ \mu_{i} & =\beta_{0}+u_{\text{[subj[i])}}+\beta_{1}\times\text{shift}_{i} \\ Anxiousness & \sim Normal\left( \mu_{i},\sigma_{e} \right) \\ Priors & : \\ \beta_{0} & \sim Normal\left( 3.5,.6 \right) \\ \beta_{1} & \sim Normal\left( 0,.5 \right) \\ u & \sim Normal\left( 0,\sigma_{u} \right) \\ \sigma_{e},\sigma_{u} & \sim Exponential\left( 1 \right) \\ & \end{matrix}$$

**rstanarm code:** stan_glmer(formula = orolig_sanggaendet ~ QR_week + (1 | id), data = d1, family = gaussian, cores = 4, prior = normal(0, 0.5, autoscale = F), prior_intercept = normal(3.5, 0.6, autoscale = F), prior_aux = exponential(1))

## **Feeling rested (utsövd)**

$$\begin{matrix} Likelihood & : \\ \mu_{i} & =\beta_{0}+u_{\text{[subj[i])}}+\beta_{1}\times\text{shift}_{i} \\ Sleep length & \sim Normal\left( \mu_{i},\sigma_{e} \right) \\ Priors & : \\ \beta_{0} & \sim Normal\left( 2.5,.5 \right) \\ \beta_{1} & \sim Normal\left( 0,0.6 \right) \\ u & \sim Normal\left( 0,\sigma_{u} \right) \\ \sigma_{e},\sigma_{u} & \sim Exponential\left( 1 \right) \\ & \end{matrix}$$

**rstanarm code:** stan_glmer(formula = utsövd ~ QR_week + (1 | id), data = d1, family = gaussian, cores = 4, prior = normal(0, 0.6, autoscale = F), prior_intercept = normal(2.5, 0.5, autoscale = F), prior_aux = exponential(1))

## **Sleepiness (KSS)**

$$\begin{matrix} Likelihood & : \\ \mu_{i} & =\beta_{0\left[ \text{TIME}\left[ i \right] \right]}+u_{\left[ \text{subj}\left[ i \right],\text{TIME}\left[ i \right] \right]}+\beta_{1}\times\text{shift}_{i}+v_{[\text{subj[i]]}} \\ Sleepiness & \sim Normal\left( \mu_{i},\sigma_{e} \right) \\ Priors & : \\ \beta_{0} & \sim Normal\left( 5,1.5 \right) \\ \beta_{1} & \sim Normal\left( 0,1 \right), \\ \sigma_{e} & \sim Exponential\left( 0.5 \right) \\ \left[ \begin{matrix} u_{1} \\ u_{2} \\ ... \\ u_{6} \\ v \end{matrix} \right] & =MVNormal\left( \begin{matrix} \left[ \begin{matrix} 0 \\ 0 \\ ... \\ 0 \\ 0 \end{matrix} \right],\Sigma_{uv} \end{matrix} \right) \\ \Sigma_{uv} & =Default priors in Rstanarm \\ & \end{matrix}$$

**Note:**
*TIME index variable*: 1 = 07h, 2 = 10h, 3 = 13h, 4 = 16h, 5 = 19h, 6 = 22h
$u$ is the by-subject varying intercepts, one for each level of the index variable (6 in total)
$v$ is the by-subject varying slope, that is the each subject’s estimated variability around the fixed-effects slope.
$\Sigma_{uv}$ is the variance-coviariance matrix.

**rstanarm code:** stan_glmer(formula = KSS ~ 0 + TID + QR_week + (0 + TID + QR_week | id), data = d_sleepy, cores = 4, prior = my_prior, prior_aux = exponential(0.5)) *my_prior:* normal(location = c(5,5,5,5,5,5,0), scale = c(1.5,1.5,1.5,1.5,1.5,1.5,1), autoscale = F)

## **Sleepiness interaction model**

$$\begin{matrix} Likelihood & : \\ \mu_{i} & =\beta_{0\left[ \text{TIME}\left[ i \right] \right]}+u_{\left[ \text{subj}\left[ i \right],\text{TIME}\left[ i \right] \right]}+\beta_{1}\times\text{shift}_{i}+\beta_{2}\times\text{TIME}_{i}\times\text{shift}_{i} \\ Sleepiness & \sim Normal\left( \mu_{i},\sigma_{e} \right) \\ Priors & : \\ \beta_{0} & \sim Default priors rstanarm \\ \beta_{1} & \sim Default priors rstanarm \\ \beta_{2} & \sim Default priors rstanarm \\ \sigma_{e} & \sim Default priors rstanarm \\ \left[ \begin{matrix} u_{1} \\ u_{2} \\ ... \\ u_{5} \\ u_{6} \end{matrix} \right] & =MVNormal\left( \begin{matrix} \left[ \begin{matrix} 0 \\ 0 \\ ... \\ 0 \\ 0 \end{matrix} \right],\Sigma_{u} \end{matrix} \right) \\ \Sigma_{u} & =Default priors in Rstanarm \\ & \end{matrix}$$

**rstanarm code:** stan_glmer(formula = KSS ~ 0 + QR_week * TID + (0 + TID | id), data = d_sleepy, cores = 4)

## **Simple attention reaction time (yRT)**

$$\begin{matrix} Likelihood & : \\ \mu_{i} & =\beta_{0\text{[TID[i]}}+u_{\text{[subj[i],TID[i]]}} \\ \frac{1000}{RT_{i}} & \sim Normal\left( \mu_{i},\sigma_{e} \right) \\ Priors & : \\ \beta_{0} & \sim Default priors rstanrm \\ \sigma_{e} & \sim Default priors rstanrm \\ \left[ \begin{matrix} u_{1} \\ u_{2} \end{matrix} \right] & =MVNormal\left( \begin{matrix} \left[ \begin{matrix} 0 \\ 0 \end{matrix} \right],\Sigma_{u} \end{matrix} \right) \\ \Sigma_{u} & =Default priors in Rstanarm \\ & \end{matrix}$$

Treatment index variable (TID): 1 = Control, 2 = QR

**rstanarm code:** stan_glmer(formula = yRT ~ 0 + TID + (0 + TID | id), data = d6.RT, family = gaussian, cores = 4, iter = 4000, QR = TRUE)

## **Simple attention interaction model**

$$\begin{matrix} Likelihood & : \\ \mu_{i} & =\beta_{0\text{[TID[i]}}+u_{\text{[subj[i],TID[i]]}} \\ \frac{1000}{RT_{i}} & \sim Normal\left( \mu_{i},\sigma_{e} \right) \\ Priors & : \\ \beta_{0} & \sim Default priors rstanrm \\ \sigma_{e} & \sim Default priors rstanrm \\ \left[ \begin{matrix} u_{1} \\ u_{2} \\ ... \\ u_{5} \\ u_{6} \end{matrix} \right] & =MVNormal\left( \begin{matrix} \left[ \begin{matrix} 0 \\ 0 \\ ... \\ 0 \\ 0 \end{matrix} \right],\Sigma_{u} \end{matrix} \right) \\ \Sigma_{u} & =Default priors in Rstanarm \\ & \end{matrix}$$

*Treatment index variable (TID):* 1 = Control morning, 2 = Control afternoon, 3 = Control evening, 4 = QR morning, 5 = QR afternoon, 6 = QR evening

**rstanarm code:** stan_glmer(formula = yRT ~ 0 + TID_int + (0 + TID_int | id), data = d6.RT_int, family = gaussian, cores = 4, iter = 4000, QR = TRUE)

## **Simple attention lapses**

$$\begin{matrix} Likelihood & : \\ logit\left( p_{i} \right) & =\beta_{0\text{[TID[i]}}+u_{\text{[subj[i],TID[i]]}} \\ Lapses_{i} & \sim Binomial\left( n_{i},p_{i} \right) \\ Priors & : \\ \beta_{0} & \sim Normal\left( -2.5,1 \right) \\ \left[ \begin{matrix} u_{1} \\ u_{2} \end{matrix} \right] & =MVNormal\left( \begin{matrix} \left[ \begin{matrix} 0 \\ 0 \end{matrix} \right],\Sigma_{u} \end{matrix} \right) \\ \Sigma_{u} & =Default priors in Rstanarm \\ & \end{matrix}$$

Treatment index variable (TID): 1 = Control, 2 = QR

**Rstanarm code:** stan_glmer(formula = lapse ~ 0 + TID + (0 + TID | id), data = d6_lapses, family = binomial(link = “logit”), cores = 4, prior = normal(-2.5, 1, autoscale = F))

## **Episodic memory, probability of misremembering words (mistake)**

$$\begin{matrix} Likelihood & : \\ logit\left( p_{i} \right) & =\beta_{0\text{[TID[i]}}+u_{\text{[subj[i],TID[i]]}} \\ Mistakes_{i} & \sim Binomial\left( n_{i},p_{i} \right) \\ Priors & : \\ \beta_{0} & \sim Normal\left( -2,1 \right) \\ \left[ \begin{matrix} u_{1} \\ u_{2} \end{matrix} \right] & =MVNormal\left( \begin{matrix} \left[ \begin{matrix} 0 \\ 0 \end{matrix} \right],\Sigma_{u} \end{matrix} \right) \\ \Sigma_{u} & =Default priors in Rstanarm \\ & \end{matrix}$$

Treatment index variable (TID): 1 = Control, 2 = QR

**Rstanarm code:** stan_glmer(formula = mistake ~ 0 + TID + (0 + TID | id), data = d7, family = binomial(link = “logit”), cores = 4, prior = normal(-2, 1, autoscale = F))

## **Episodic memory interaction**

$$\begin{matrix} Likelihood & : \\ logit\left( p_{i} \right) & =\beta_{0\text{[TID[i]}}+u_{\text{[subj[i],TID[i]]}} \\ Mistakes_{i} & \sim Binomial\left( n_{i},p_{i} \right) \\ Priors & : \\ \beta_{0} & \sim Normal\left( -2,1 \right) \\ \left[ \begin{matrix} u_{1} \\ u_{2} \\ u_{3} \\ u_{4} \end{matrix} \right] & =MVNormal\left( \begin{matrix} \left[ \begin{matrix} 0 \\ 0 \\ 0 \\ 0 \end{matrix} \right],\Sigma_{u} \end{matrix} \right) \\ \Sigma_{u} & =Default priors in Rstanarm \\ & \end{matrix}$$

*Treatment index variable (TID):* 1 = Control morning, 2 = control afternoon, 3 = QR morning, 4 = QR afternoon

**Rstanarm code:** stan_glmer(formula = mistake ~ 0 + TID_int + (0 + TID_int | id), data = d7_int, family = binomial(link = “logit”), cores = 4, prior = normal(-2, 1, autoscale = F))

## **Stroop reaction time during incongruent and congruent trials respectively (yRT)**

$$\begin{matrix} Likelihood & : \\ \mu_{i} & =\beta_{0\text{[TID[i]}}+u_{\text{[subj[i],TID[i]]}} \\ \frac{1000}{RT_{i}} & \sim Normal\left( \mu_{i},\sigma_{e} \right) \\ \beta_{0} & \sim Default priors rstanrm \\ \sigma_{e} & \sim Default priors rstanrm \\ \left[ \begin{matrix} u_{1} \\ u_{2} \end{matrix} \right] & =MVNormal\left( \begin{matrix} \left[ \begin{matrix} 0 \\ 0 \end{matrix} \right],\Sigma_{u} \end{matrix} \right) \\ \Sigma_{u} & =Default priors in Rstanarm \\ & \end{matrix}$$

Treatment index variable (TID): 1 = Control, 2 = QR

**Rstanarm code:** stan_glmer(formula = yRT ~ 0 + TID + (0 + TID | id), data = d_incongruent, family = gaussian, cores = 4, iter = 4000, QR = TRUE)

## **Stroop incongruent trials, interaction model**

$$\begin{matrix} Likelihood & : \\ \mu_{i} & =\beta_{0\text{[TID[i]}}+u_{\text{[subj[i],TID[i]]}} \\ \frac{1000}{RT_{i}} & \sim Normal\left( \mu_{i},\sigma_{e} \right) \\ \beta_{0} & \sim Default priors rstanrm \\ \sigma_{e} & \sim Default priors rstanrm \\ \left[ \begin{matrix} u_{1} \\ u_{2} \\ .. \\ u_{5} \\ u_{6} \end{matrix} \right] & =MVNormal\left( \begin{matrix} \left[ \begin{matrix} 0 \\ 0 \\ 0 \\ 0 \end{matrix} \right],\Sigma_{u} \end{matrix} \right) \\ \Sigma_{u} & =Default priors in Rstanarm \\ & \end{matrix}$$

*Treatment index variable (TID):* 1 = Control morning, 2 = Control afternoon, 3 = Control evening, 4 = QR morning, 5 = QR afternoon, 6 = QR evening

**rstanarm code:** stan_glmer(formula = yRT ~ 0 + TID_int + (0 + TID_int | id), data = d8a_interact, family = gaussian, cores = 4, iter = 4000, QR = TRUE)

## **Stroop probability of mistakes**

$$\begin{matrix} Likelihood & : \\ logit\left( p_{i} \right) & =\beta_{0\text{[TID[i]}}+u_{\text{[subj[i],TID[i]]}} \\ Misses_{i} & \sim Bernoulli\left( N_{i},p_{i} \right) \\ Priors & : \\ \beta_{0} & \sim Normal\left( -2,1 \right) \\ \left[ \begin{matrix} u_{1} \\ u_{2} \end{matrix} \right] & =MVNormal\left( \begin{matrix} \left[ \begin{matrix} 0 \\ 0 \end{matrix} \right],\Sigma_{u} \end{matrix} \right) \\ \Sigma_{u} & =Default priors in Rstanarm \\ & \end{matrix}$$

Treatment index variable (TID): 1 = Control, 2 = QR

**rstanarm code:** stan_glmer(formula = mistake ~ 0 + TID + (0 + TID | id), data = d8_mistakes, family = binomial(link = “logit”), iter = 4000, cores = 4, prior = normal(-2, 1, autoscale = F))

## **Stroop Congruency effect**

The congruency effect was operationalized as the difference in reaction time between cI and cC trials

$$\begin{matrix} Likelihood & : \\ \mu_{i} & =\beta_{0}+u_{0\text{[subj[i]]}}+\beta_{1}\times\text{Shift}_{i}+u_{1\text{[subj[i]]}}+\beta_{2}\times\text{Conflict}_{i}+u_{2\text{[subj[i]]}} +\beta_{3}\times\text{Shift}_{i}\times\text{Conflict}_{i}+u_{3\text{[subj[i]]}} \\ \frac{1000}{RT_{i}} & \sim Normal\left( \mu_{i},\sigma_{e} \right) \\ Priors & : \\ \beta_{0} & \sim default \\ \beta_{1} & \sim default \\ \beta_{2} & \sim default \\ \beta_{3} & \sim default \\ \sigma_{e} & \sim default \\ \left[ \begin{matrix} u_{0} \\ u_{1} \\ u_{2} \\ u_{3} \end{matrix} \right] & =MVNormal\left( \begin{matrix} \left[ \begin{matrix} 0 \\ 0 \\ 0 \\ 0 \end{matrix} \right],\Sigma_{u} \end{matrix} \right) \\ \Sigma_{u} & =Default priors in Rstanarm \\ & \end{matrix}$$

$\beta_{0}$ = represents cC trials during day transitions

$\beta_{0}+\beta_{1}$ = represents cC trials during quick returns transitions.

$\beta_{0}+\beta_{2}$ = represents cI trials during day-transitoons.

$\beta_{0}+\beta_{1}+\beta_{2}+\beta_{3}$ = represents cI trials during quick returns.

**rstanarm code:** stan_glmer(formula = yRT ~ conflict * QR_week + (conflict * QR_week | id), data = d8_cc, family = gaussian, cores = 4, iter = 6000, QR = T)

##

## **Subjective ratings of cognitive performance and safety (PC1)**

$$\begin{matrix} Likelihood & : \\ \mu_{i} & =\beta_{0}+u_{\text{[subj[i]]}}+\beta_{1}\times Shift_{i} \\ Y_{i} & \sim Normal\left( \mu_{i},\sigma_{e} \right) \\ Priors & : \\ \beta_{0} & \sim default \\ \beta_{1} & \sim default \\ u & \sim default \\ \sigma_{e},\sigma_{u} & \sim default \\ & \end{matrix}$$

**rstanarm code:** stan_glmer(formula = PC1 ~ QR_week + (1 | id), data = d_pca1, family = gaussian, cores = 4)

# Deviations from the pre-registration

## **Additional secondary outcomes**

Sleep efficiency and the Karolinska Sleep Diary – Sleep Quality Index (KSD-SQI) were added as additional secondary outcomes of sleep quality.

## **Cut offs**

For reaction time test, we had planned to use 3x the individual standard deviation on the 1000/RT (1RT) scale, as reaction times aren’t normally distributed on the original scale. Lower values on the 1RT-scale corresponds to longer reaction times. Subtracting 3xsd from the mean response speed (1RT) sometimes result in negative response times. Thus, the upper bound of outliers (exceptionally long reaction times) became undefined for some indiduals. Defining outliers as 3x the standard deviation on the original scale on the other hand, resulted response times < 1000 ms to be defined as outliers (upper bound), which is more than three times faster than the cut off of 3000ms that was used in Holding et al (2021). Therefor, we adjusted the cut off for outliers to responses <150ms and >3000ms.

The model estimates were slightly affected but it did not impact our interpretations of the results, as the compatibility interval clearly overlapped zero for all models.

- Pre-registered cut off ($\pm3SD$): estimated mean difference= -6, 95 % CI [-17, 5]
- Revised cut off: estimated mean difference = -9, 95 % CI [-20, 3]
- Pre-registered cut off ($\pm3SD$), day 3: estimated mean difference = -10, 95 % CI [-27, 6]
- Revised cut off, day 3: estimated mean difference = -12, 95 % CI [-30, 5]

## **Model parameterisations deviating from pre-registration:**

- The intended TID-paremarisation would have allowed us to fit a single model, and extract both main effects and interaction effects. As explained below, we had to deviate from this plan and specify the interaction models separately:
  - For sleepiness, each TID had exactly one observation, and thus a random structure with one random effect per TID-level was unidentifiable. Thus, a dummy-parameterization was used with a random intercept only (see section with model specifications)
  - For the cognitive tests, participants were instructed to take the cognitive tests at 08:00-10:00 (morning), after the shift ended (afternoon), prior to going to bed (evening). However, the actual times when the tests were taken varied greatly. For example, the morning test was sometimes taken at noon, and evening tests taken as early as 18:00. Therefor, the TID-parameterisation could not be based on the test number as intended, but had to be determined based on the actual time for taking the test. In addition, we needed to make sure that there was sufficient contrast between the three tests. Thus, the TID-parameters were defined as follows: morning tests (≤ 10:00), afternoon tests (≥12:00 & ≤17:00) and evening tests (≥ 18:30). As these definitions meant that some data was lost, the parameterisation was only used for the interaction model and not the models of the main analysis. For the main analysis, we only estimated differences between conditions.
- For episodic memory, we had planned to use Binomial(48,$p_{i}$), but instead used Binomial(n=1,$p_{i}$), as each of the 48 cases were stored as a separate data point in the data file. These two parameterisations can be used interchangeably.
- For reaction time, we had incorrectly speficied response speed as 1/reaction time, when it should be 1000/reaction time as reaction times are recorded in milliseconds.
- Due to convergence issues, the model of cognitive conflict was specified using a dummy-parameterisation instead, see section on model specifications.

## **Priors deviating from pre-registration**

- For the interaction model of sleepiness, default rstanarm priors were used, as we changed the paramterization (see above).
- In the pre-registration, we also specified priors for informed models that could be used to aggregate knowledge across studies by utilising Bayesian updating. However, these models are less conservative than the weakly informed models as they intentionally bias estimates toward previously observed effect sizes and thus were not intended for hypothesis testing. Due to limited space, only the data from the weakly regularised models are included in the paper. The model estimates with informed priors can be acquired by contacting the corresponding author of the paper.
